# Dual energy CT in necrotizing enterocolitis; a novel diagnostic approach

**DOI:** 10.3906/sag-2103-294

**Published:** 2021-10-21

**Authors:** Özgür ÇAĞLAR, Emrullah CESUR, Recep SADE, Binali FIRINCI, Mustafa KARA, Mehmet Emin ÇELİKKAYA, Akgün ORAL, Murat YİĞİTER, Sevilay ÖZMEN

**Affiliations:** 1 Department of Pediatric Surgery, Faculty of Medicine, Atatürk University, Erzurum Turkey; 2 Department of Radiology, Faculty of Medicine, Atatürk University, Erzurum Turkey; 3 Department of Neonatology, Faculty of Medicine, Atatürk University, Erzurum Turkey; 4 Department of Pediatric Surgery, Faculty of Medicine, Mustafa Kemal University, Hatay Turkey; 5 Department of Pediatric Surgery, S.B.U. Dr. Behçet Uz Children’s Education and Research Hospital, İzmir Turkey; 6 Department of Pathology, Faculty of Medicine, Atatürk University, Erzurum Turkey

**Keywords:** Dual-energy computed tomography, necrotizing enterocolitis, newborn

## Abstract

**Background/aim:**

Necrotizing enterocolitis (NEC) is one of the most important causes of morbidity and mortality in premature infants. Although there are no specific diagnostic tools, the main factors affecting prognosis are clinical and laboratory findings, and early diagnosis and treatment. In this study, we demonstrate the importance of dual-energy computed tomography (DECT) in confirming intestinal ischemia in neonates with NEC.

**Materials and methods:**

Patients diagnosed with NEC in a neonatal intensive care unit were staged according to modified Bell’s classification, and DECT was performed on patients with NEC stages 2-A, 2-B and 3-A. According to their DECT reports, these patients were then separated into two groups: those with intestinal ischemia and those without intestinal ischemia. The patients with intestinal ischemia were evaluated using surgical reports, and the other patients were evaluated using clinical findings.

**Results:**

DECT was performed in 21 patients with NEC stages 2-A, 2-B and 3-A. Twelve patients (57.1%) without ischemia were followed up without surgery. Nine patients (42.9%) with ischemia on DECT were operated on, and resection and anastomosis or ileostomy and colostomy were performed.

**Conclusion:**

In patients with NEC, DECT significantly increases overall diagnostic confidence in assessing intestinal necrosis when compared with traditional diagnostic methods.

## 1. Introduction

NEC is one of the most important causes of morbidity and mortality in neonatal intensive care units [1–3]. It affects 4%–13% of infants have low birth weight (<1500 g) and 1%–8% of infants admitted to neonatal intensive care units [4, 5]. Clinical findings are manifested in a variety of ways, including feed intolerance, prefeed residue with bile-stained aspirates and/or vomit, bradycardia, fulminant shock, and death. Plain antero-posterior and left lateral decubitis radiographs are the cornerstone of NEC diagnosis. According to clinical severity, pneumatosis intestinalis (PI), portal vein gas (PVG), pneuomoperitoneum (PP), intraperitoneal fluid, and persistently dilated fixed loops are some of the associated findings can be seen on plain abdominal graphy (PAG) [6]. Ideally, surgery should not be performed until gangrene is present; however, it should be undertaken before perforation occurs.

Dual-energy computed tomography (DECT) is a new technique that has the capability of differentiating matter and tissue according to CT density values obtained from two synchronous CT acquisitions at different tube potentials. It should be emphasized that described systems can also be used for simply clinical routine examination requiring standard single-energy mode DECT acquisition [7]. Dual-source CT system is used for the evaluation of renal masses, liver lesions, urinary calculi, small bowel, pancreas, and adrenal glands [8–11]. This study is the first report on the use of DECT as a diagnostic modality in patients with NEC. 

DECT will enable better detection of hypovascular segments of the bowel that enhance poorly. DECT makes it possible to distinguish different types of matter that cause high-density areas in and around the bowel. In this study, we aimed to detect intestinal ischemia in newborns with NEC using a novel method. Therefore, we think that surgical decision-making processes of these patients before bowel perforation may be easier and may provide positive contributions to patients in terms of morbidity and mortality.

## 2. Material and methods 

Over a period of 36 months, 34 consecutive patients with a clinical diagnosis of NEC were enrolled in this observational study. The study was approved by the medical ethics committee of our hospital (03.12.2015/10). 

The clinical and laboratory findings were recorded. The initial diagnosis of NEC was clinical and based mainly on signs and symptoms such as abdominal distension, gastric residue, vomiting (feeding intolerance), lack of stool, diarrhea, hematochezia, abdominal wall erythema, and palpable abdominal mass. All patients were assessed using plain abdominal radiography, and the radiographs were explicated by pediatric radiologists. The radiological criteria evaluated as predictors of NEC on plain abdominal radiographs were PI, PVG, PP, fixed intestinal loops, gasless abdomen, and peritoneal fluid. 

Modified Bell’s classification was used to determine the stage of the disease [4]. Each patient was scanned with DECT, except for those whose clinical condition made it difficult to transport them to the radiology unit and those classified as Bell’s stage 1-A, 1-B, 3-B, since it is unacceptable to expose newborns to radiation unnecessarily. DECT results were recorded as showing necrosis to be present or not present. 

Indications for surgical treatment were clinical or radiological evidence of intestinal perforation, abdominal wall erythema/edema indicating gangrene, presence of abdominal palpable mass, failure to respond to conservative management with a rapid decline in health status, and necrosis on DECT (regardless of whether the intestinal perforation was confirmed by other radiological examinations).

**Figure 1:**
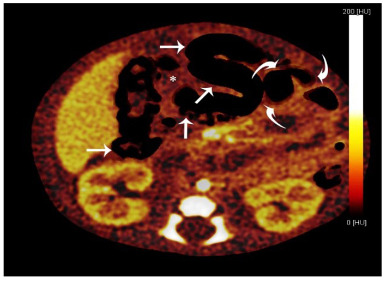
The iodine map of a ten-day-old male newborn showing dark mesenteric areas (see asterisk), indicating unenhanced areas. The ischemic intestinal walls were also dark (see arrow), and the normal intestinal walls were bright (see curved arrows).

The DECT examinations were carried out by using a 256-slice dual-source CT (Somatom Definition Flash, Siemens Healthcare, Forchheim, Germany). Iopromide (2 mL × kg, Ultravist 300, 300 mg/mL, Bayer Schering Pharma, Berlin, Germany) followed by 30 mL of saline was injected into the antecubital vein at a flow rate of 3 mL/s. A bolus tracking technique was used, and the region of interest was located in the abdominal aorta. The following scanning protocols were carried out: tube voltage of 100 kVp/Sn 140 kV (140 kV with tin filter); automatic mAs current (by CARE Dose4D); 64 × 0.6 mm collimation; rotation time of 0.28 seconds; automatic pitch; cranio-caudal scan direction; slice width 0.6 mm; increment 0.3 mm; kernel D33f abdominal view; and temporal resolution of 75/140/330 ms. The CT dose index volume and dose length product of the DECT scans were recorded for each patient protocol. High or low voltage data were reconstructed separately to analyze of abdominal perfusion. To optimize signal/noise ratio, dual-energy convolution kernel (D30f) is used, which is composed of 140 ms temporal resolution with 1.5 mm thickness and 1 mm increments. A specific platform (syngo Multi-Modality Workplace; Siemens, Erlangen, Germany) was used for extended analysis of the reconstructed data sets. Tissue data analyzed with 0.6 mm same sections reconstructed with 330 ms temporal resolution, dedicated algorithm of each abdominal perfusion blood volume for the evaluation of abdominal perfusion incorporation obtaining dual-energy image of post processing software application (Multi-Modality Workplace-syngo Dual Energy/Siemens). Two radiologists (M. K. and R. S., who have six and three years of experience with DECT, respectively) were blinded to the clinical data. They examined the CT images, and iodine map images were obtained from 21 examinations independently; in case of disagreement, a final decision was reached by consensus. The dark areas on the iodine map were considered to be necrotic (Figure 1).

The data were analyzed using SPSS 15.0 software, and the Kolmogorov–Smirnov test was applied. Percentages, mean values, and standard deviations were used as identifier statistical values. Specificity, sensitivity, positive/negative predictive values, and diagnostic accuracy rates were calculated for clinical and radiological criteria, and statistical significance was observed for diagnostic tests using standard epidemiological measures. Patients who received surgery were evaluated in terms of surgical outcomes, while nonsurgical patients were evaluated in terms of clinical results.

## 3. Results

Data were analyzed from 34 patients (16 males and 18 females) with a diagnosis of NEC who were followed between October 2015 and October 2018. The mean birth weeks of these patients were 30.12+/−3.98 weeks, and their mean birth weights were 1306.21+/−527.46 grams. Five patients had stage 1 NEC (2 had 1-A, 3 had 1-B), 22 patients had stage 2 NEC (11 had 2-A, 11 had 2-B), and seven patients had stage 3 NEC (5 had 3-A, 2 had 3-B) according to modified Bell’s classification (Table 1). The clinical and laboratory findings of the patients were recorded. The clinical and laboratory findings are shown in Table 1.

**Table 1:** Demographic, clinical, laboratory and radiologic findings of study.

Gender (F/M) n (%)		18 (52.9) / 16 (47.1)
Birth week		30.12 ± 3.98
Weight (g)		1306.21 ± 527.46
Bells’ stage n (I/II/III)		5/ 22 / 7
I-A/I-B		2/3
II-A/II-B		11/11
III-A/III-B		5/2
	Abdominal skin erythema	5
	Rectal bleeding	4
	Palpabl abdominal mass	3
	Neutropenia	11
	Trombocytopenia	15
	Metabolic failure	13
Radiologic findings		
	Dilated intestinal loop	27
	Fixed intestinal loop	11
	Pneumotisis intestinalis	16
	Portal vein gas	10

All values given as mean±SD, n or %. F: Female, M: Male.

 DECT was not performed to patients with stage 3-B and all stage 1 NEC patients. 

**Figure 2:**
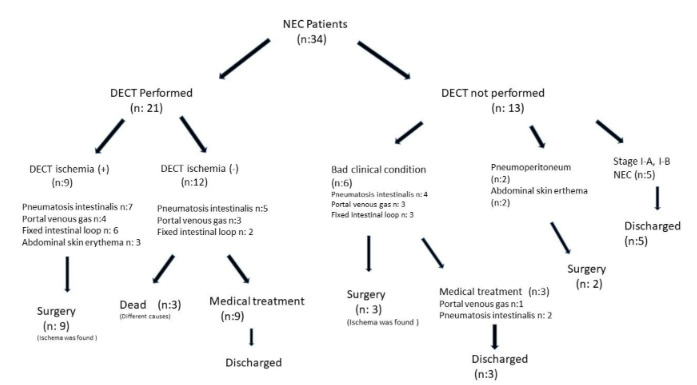
Flow chart of treatment and outcomes for the 34 patients presenting with NEC.

**Figure 3:**
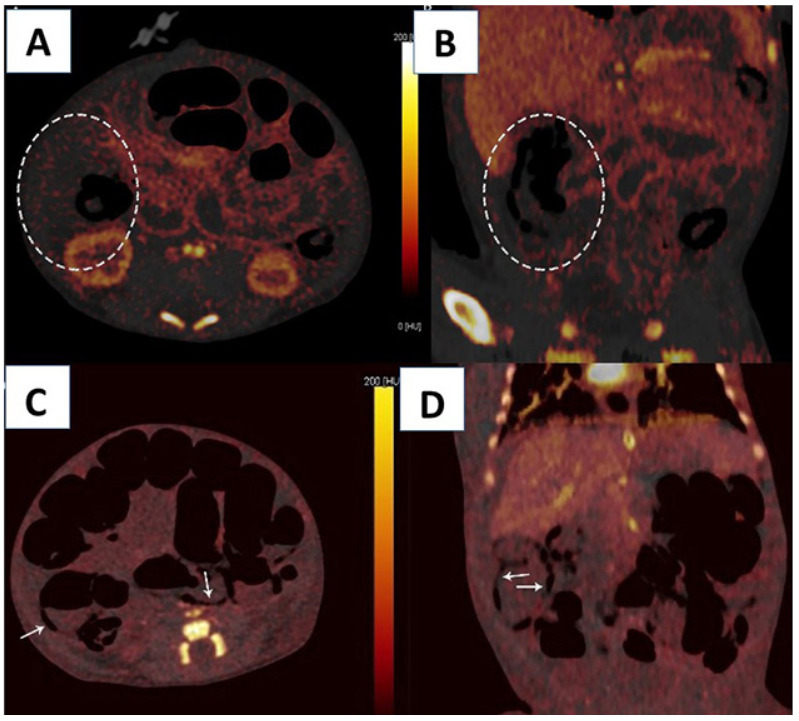
Axial (A) and coronal (B) iodine map images of an infant showing ischemic bowel and mesentery (see circled area). The other parts of the bowel and mesentery are colored with iodine contrast. Axial (C) and coronal (D) iodine map images of an infant showing wide ischemic bowel and mesentery. PI is also present (see arrows).

**Figure 4:**
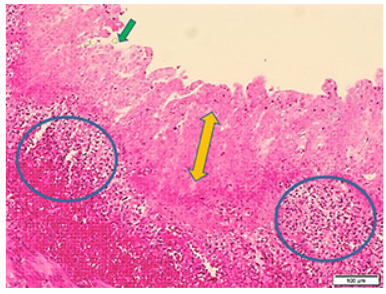
The histopathological view of intestinal wall characterized with t ransmucosal necrosis (yellow arrow) including spread inflammatory infiltration (circles), surface epithelium necrosis, and villus atrophy (green arrow).

DECT was performed in 21 patients with stages 2-A (9 patients), 2-B (9 patients) and 3-A (3 patients) (Figure 2). Ischemia was detected in nine patients (3 had stage 2-A, 4 had stage 2-B and 2 had stage 3-A) using DECT (Figures 3A, B, C, D) and confirmed at surgery in all of these patients. Seven patients had PI, four had PVG, six had fixed intestinal loop on PAG. Three patients had abdominal skin erythema (Figure 2). Ostomy or end-to-end anastomosis was performed after resection for ischemic segments at operation. Histologic assessment of the resected specimens confirmed bowel ischemia in all (Figure 4). Our study found DECT to have 100% sensitivity and 100% specificity for detecting bowel ischemia and 100% positive predictive value (Table 2). 

**Table 2:** Sensitivity and specificity of DECT, PI, PVG on detecting intestinal ischemia.

	DECT	PI	PVG
Sensitivity	%100	%61.5	%38.5
Specificity	%100	%57.1	%76.2
Positive predictive value	%100	%47.1	%50
Negative predictive value	%100	%70.6	%66.7
Diagnostic accuracy	1	0.62	0.65

DECT: dual-energy computed tomography, PI: pneumatosis intestinalis, PVG: portal venous gas.

**Figure 5:**
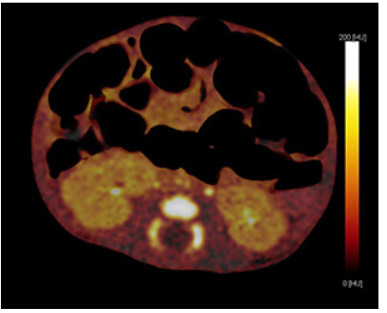
Iodine map images showing normal bright mesentery and intestinal walls.

**Figure 6:**
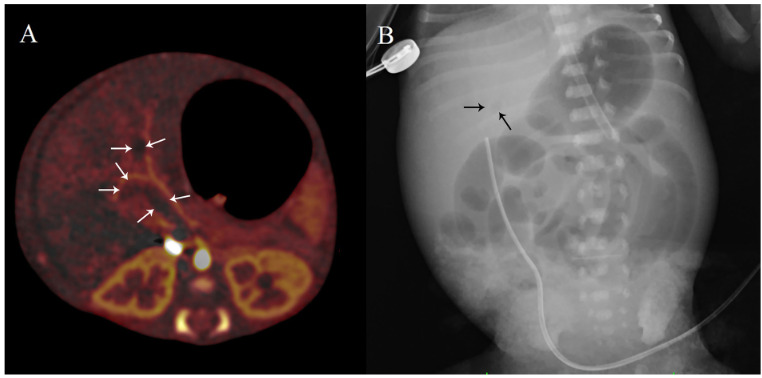
Axial contrast-enhanced image (A) of a preterm infant showing portal thrombus (see white arrows). The radiolucent area (see black arrows) on the direct abdominal X-ray (B) was misdiagnosed as PVG.

Bowel ischemia was not detected in 12 patients (6 had stage 2-A, 5 had stage 2-B, and 1 had stage 3-A) on DECT (Figure 5). Five of these patients had PI, three had PVG, and two had fixed intestinal loop on PAG (Figure 2). A patient who had PVG on PAG was later verified on DECT as having portal venous thrombus (Figures 6A, 6B). Nine of these 12 patients were discharged uneventfully. One patient who received treatment for portal venous thrombus died of pulmonary and intracranial hemorrhage, another died of cardiac pathologies (wide PDA, ASD, and hypertrophied cardiomyopathy), and another died of pulmonary hemorrhage related to respiratory distress syndrome.

We calculated the following statistical values for PI: sensitivity 61.5%, specificity 57.1%, positive predictive value 47.1%, negative predictive value 70.6%, and diagnostic accuracy 0.62. We calculated the following statistical values for PVG: sensitivity 38.5%, specificity 76.2%, positive predictive value 50.0%, negative predictive value 66.7%, and diagnostic accuracy 0.65 (Table 2).

The effective radiation dose of DECT was calculated using a method proposed by the American Association of Physics in Medicine for the measurement, reporting, and management of radiation doses in CT. Using the dose length product and a conversion coefficient for the neonate abdomen (k = 0.049 mSv/[mGy cm]), the mean effective radiation dose for DECT was calculated as 2.21 ± 0.24 mSv.

## 4. Discussion

NEC is one of the most common and most serious acquired bowel diseases in premature newborns. Over the past 20 years, great improvements in neonatal medicine have resulted in improved results. In spite of the decrease of NEC morbidity and mortality, the incidence of NEC is increasing [12]. Early diagnosis and treatment are the main factors affecting prognosis; in particular, diagnosis of ischemia in clinically suspected patients is extremely important in improving mortality rates [6,13]. 

Today’s practice is limited to a few nonspecific markers of systemic disease (e.g., platelets, C‑reactive protein, and white blood cells). Although most of the physicians have tried to understand the etiology of NEC for early diagnosis and effective treatment; immunological markers of the disease should also be examined [14]. Evenett et al. conducted a systematic review of previous studies of serological tests to establish which of them are useful in the diagnosis of NEC [15]. Their findings suggested that intestinal fatty-acid binding protein and platelet‑activating factor have sensitivity and specificity in the diagnosis of NEC. Nevertheless, for more reliable results, these indicators should be tested in further prospective studies with larger sample groups [15].

Most surgeons rely on radiological signs to establish a diagnosis of NEC. Plain radiographs are essential for the radiological diagnosis of NEC, but the specificity and sensitivity of this method are relatively weak. PI (cystic or linear) is the most specific radiographic finding. Other common radiographic findings are gas-filled loops of bowel, persistently dilated loops of bowel, air-fluid levels, thickened bowel walls, PVG, and PP. PI is a pathognomonic sign of NEC in a true clinical setting. However, the level of PI does not correspond in every case with the severity of the disease [16–18], and Alda and colleagues reported the sensitivity of PI findings as lower than 50% [6]. In our study, DECT was performed in 12 of 16 patients with PI. Intestinal ischemia was not determined in five of these 12 patients, and four of the five were discharged without any problems. A 25-week-old patient died of cardiac and respiratory failure.

PVG, another hallmark of NEC, is a less common radiographic sign associated with higher mortality rates and severe disease [16,19]. Nonetheless, with medical management, many patients with PVG recover fully [20]. Karl Muchantef and colleagues commented on sonographic and radiologic images for 55 patients, and they did not accept PVG and intramural gas as absolute surgical indications [21]. The results of the present study are in line with these observations, showing that air in the portal system was the variable associated with a high risk of intestinal necrosis. DECT was performed in seven of the 10 patients, and intestinal necrosis was found in only four of them. PVG had a positive predictive value of 50%, a negative predictive value of 66.7%, and a diagnostic accuracy rate of 0.65.

The presence of PP on radiograph is accepted as an absolute indication for surgery [6, 22]. However, PP has been reported in approximately 50% to 75% of patients with perforation [6, 23]. In cases where clinicians wait for evidence of PP before deciding to perform surgery, it may be far too late for the patient, especially for an extremely premature infant.

Laparoscopy was used as a method to identify necrotic bowel segments and to decide requiring in NEC [24,25]. It has been experimentally shown that fluorescein laparoscopy is superior to another diagnostic methods to identify early stage of NEC and to distinguish reversible and irreversible intestinal ischemia [26–28]. Recently, Numanoğlu and colleagues have emphasized that this method increases diagnostic accuracy rate of intestinal ischemia in suspected NEC patients [29]. However, this study was performed on only 13 patients, and laparotomy was performed in 10 of these patients due to detected intestinal ischemia. Negative laparoscopy does not seem to be acceptable in patients undergoing anesthesia, such as suspected NEC, in patients whose general condition is extremely impaired and other system anomalies are frequently observed.

On conventional CT, it is not generally possible to appreciate the signs of reduced or absent mural enhancement in cases of intramural hemorrhage or early ischemia, or when mural enhancement is suboptimal because of high image noise or low IV contrast concentration. It has been suggested that CT, contrast examinations of the gastrointestinal tract, and magnetic resonance imaging are not clinically useful modalities for the treatment of infants with NEC [20]. The most important reasons for this are the high radiation rates of CT and the frequent possibility of diagnosing NEC by means of physical examination and other modalities. According to the literature, the mean radiation dose for CT for pediatric patients is between 5.7 and 6.2 mSv [30]. Our radiation dose (2.21 ± 0.24 mSv) is lower than literature and equivalent to about 20 plain radiographs [30]. Although our doses are within normal limits.

It has been shown that DECT mostly specific to detect of hypovascular solid organ lesions such as liver and pancreas. In this respect, it is thought that this technique will enable better detection of hypovascular segments of the bowel that enhance poorly. It also has the ability to emphasize mural hyper-enhancement or hypo-enhancement. Primary preclinical studies have indicated that DECT has significant potential to describe intestinal infarction [31]. Moreover, when ischemia in small intestinal obstruction is evaluated using low‑keV virtual monoenergetic images, early clinical results have suggested an increase in diagnostic confidence [32]. Subsequently, Lourenco has reported that iodine map and 40 keV monoenergetic images increase the conspicuity of acute intestinal ischemia, resulting in improved diagnostic accuracy [33]. The use of low-keV virtual mono-energetic images mostly detect increased vascularization of the inflamed intestinal wall such as Crohn’s disease [32]. As active peristalsis of the bowel mistakenly considered as wall hyperemia on CT imaging, iodine density images should be used to eliminate of artefacts [34]. However, as yet there has been no study in English of DECT and its beneficial effects in cases of NEC.

In this study, we found that all patients with intestinal ischemia on DECT also had intestinal ischemia at surgery. The patients without ischemia on DECT examination were treated medically, and nine of them were discharged without any problems. Three patients died of nongastrointestinal causes. We found that DECT had a diagnostic accuracy rate of 100% for bowel ischemia. 

There are some limitations in this study. It should be borne in mind that infants with advanced NEC almost always need mechanical ventilator support and high levels of inotropic support. For that reason, in a hospital where the radiological unit is situated some distance from the intensive care unit, transportation of such patients for DECT observation may be highly problematic, and serious difficulties may be experienced. Even if the radiological unit is close to the intensive care unit, significant clinical deterioration may occur during the transportation of an intubated infant under inotropic support. Moreover, it is very well known that some patients may have transient intestinal ischemia that resolves with bowel rest and antibiotic therapy (unfortunately, no one knows the natural history of bowel ischemia). On the other hand, we also know that mild ischemia may only involve the mucosa and that it takes more to cause full-thickness necrosis. In this clinical study, we have no evidence that the study can quantitate the degree of ischemia. So, we need to design a study in rats by using a NEC model in which different degrees of ischemia could be induced. Only, with such a study, we can know whether DECT can demonstrate the presence of a mild ischemia and whether these infants with DECT positive for mucosal ischemia would have got away without surgery. 

## 5. Conclusions 

In NEC patients, the use of DECT may help to improve early detection of bowel ischemia by increasing the conspicuity of under perfused tissues on low-kiloelectron-volt and iodine material density images. Additional investigation of DECT must be carried out in larger series to evaluate its potential to improve the sensitivity and specificity of conventional radiological methods for detecting early small bowel ischemia.

## Funding information

The authors declare that no funding was received.

## Ethical approval

The study was approved by the medical ethics committee of our hospital (03.12.2015/10). All procedures involving human beings were conducted following the ethical standards and principles outlined in the Helsinki Declaration (2008).
